# Transcriptome Analysis Suggests That Chromosome Introgression Fragments from Sea Island Cotton (*Gossypium barbadense*) Increase Fiber Strength in Upland Cotton (*Gossypium hirsutum*)

**DOI:** 10.1534/g3.117.300108

**Published:** 2017-09-05

**Authors:** Quanwei Lu, Yuzhen Shi, Xianghui Xiao, Pengtao Li, Juwu Gong, Wankui Gong, Aiying Liu, Haihong Shang, Junwen Li, Qun Ge, Weiwu Song, Shaoqi Li, Zhen Zhang, Md Harun or Rashid, Renhai Peng, Youlu Yuan, Jinling Huang

**Affiliations:** * College of Agriculture, Shanxi Agricultural University, Taigu 030801, Shanxi, China; † State Key Laboratory of Cotton Biology, Key Laboratory of Biological and Genetic Breeding of Cotton, The Ministry of Agriculture, Institute of Cotton Research, Chinese Academy of Agricultural Sciences, Anyang 455000, Henan, China; ‡ School of Biotechnology and Food Engineering, Anyang Institute of Technology, Anyang 455000, Henan, China

**Keywords:** cotton, fiber strength, transcriptome, DEG, secondary cell wall synthesis, GenPred, Shared Data Resources, Genomic Selection

## Abstract

As high-strength cotton fibers are critical components of high quality cotton, developing cotton cultivars with high-strength fibers as well as high yield is a top priority for cotton development. Recently, chromosome segment substitution lines (CSSLs) have been developed from high-yield Upland cotton (*Gossypium hirsutum*) crossed with high-quality Sea Island cotton (*G. barbadense*). Here, we constructed a CSSL population by crossing CCRI45, a high-yield Upland cotton cultivar, with Hai1, a Sea Island cotton cultivar with superior fiber quality. We then selected two CSSLs with significantly higher fiber strength than CCRI45 (MBI7747 and MBI7561), and one CSSL with lower fiber strength than CCRI45 (MBI7285), for further analysis. We sequenced all four transcriptomes at four different time points postanthesis, and clustered the 44,678 identified genes by function. We identified 2200 common differentially-expressed genes (DEGs): those that were found in both high quality CSSLs (MBI7747 and MBI7561), but not in the low quality CSSL (MBI7285). Many of these genes were associated with various metabolic pathways that affect fiber strength. Upregulated DEGs were associated with polysaccharide metabolic regulation, single-organism localization, cell wall organization, and biogenesis, while the downregulated DEGs were associated with microtubule regulation, the cellular response to stress, and the cell cycle. Further analyses indicated that three genes, XLOC_036333 [mannosyl-oligosaccharide-α-mannosidase (*MNS1*)], XLOC_029945 (*FLA8*), and XLOC_075372 (*snakin-1*), were potentially important for the regulation of cotton fiber strength. Our results suggest that these genes may be good candidates for future investigation of the molecular mechanisms of fiber strength formation and for the improvement of cotton fiber quality through molecular breeding.

Cotton is one of the largest commercial fiber crops worldwide, and represents the most prevalent source of renewable natural fiber for the global textile industry (Wendel and Cronn 2003). The simultaneous improvement of fiber quality and yield is currently the primary goal of cotton breeding programs (Constable *et al.* 2015). Upland cotton (*Gossypium hirsutum*) produces high yields of relatively low-quality fibers, whereas Sea Island cotton (*G. barbadense*) produces relatively low yields of high-quality fibers (Shi *et al.* 2015). Upland cotton plants are usually crossed with Sea Island cotton plants to generate introgression lines that produce improved yields of longer, stronger, finer fibers (Fang *et al.* 2014b; Gilbert *et al.* 2014).

Cotton fibers develop from single epidermal cells of the developing ovule (Gao *et al.* 2012); these epidermal cells serve as excellent models for biological studies of plant cell elongation, as well as of the mechanisms of biosynthesis of the cell wall and cellulose (Liu *et al.* 2015). From initiation [∼3 d postanthesis (DPA)] until 23 DPA, the fiber cells in *G. hirsutum* rapidly elongate, with a peak elongation rate of >2 mm/d (Fang *et al.* 2014b). Overlapping with this elongation stage, from 15 to 40 DPA, fiber cells deposit the secondary wall; maturation occurs between 40 and 50 DPA (Kim and Triplett 2001).

Fiber strength is a key indicator of cotton fiber quality (Shang *et al.* 2016). Cellulose is the primary component of cotton fibers (>90%), providing not only fiber strength, but also mechanical support and protection for the plant body (Edelmann and Fry 1992). The biosynthesis of cellulose increases over 100-fold during the deposition of the secondary cell wall (Zhong and Ye 2015). Rapid and dynamic changes in gene expression during this stage indicate that an amalgamation of complex processes may contribute to cotton fiber development, including transcriptional regulation, signal transduction, and metabolic pathway induction (Pan *et al.* 2007; Kim and Triplett 2001; Tuttle *et al.* 2015).

Several of the multiple genes that are involved in the deposition of the secondary cell wall and resultant fiber strength have been identified (Z. Zhang *et al.* 2015). For example, in *G. hirsutum*, the over-expression of *GbEXPA2* produces fibers with slightly thicker walls and higher crystalline cellulose content, while the overexpression of *GbEXPATR* produces longer, finer, stronger fibers with significantly thinner cell walls (Y. Li *et al.* 2016).

In addition to secondary cell wall biosynthesis, hormone responses might also influence fiber strength by improving fiber-to-fiber interactions (Islam *et al.* 2016a): receptor-like kinase signaling pathways might regulate cotton-fiber cell-wall assembly and strength by coordinating cell elongation and secondary cell wall biosynthesis (Islam *et al.* 2016a). However, the molecular mechanisms that regulate the strength and formation of the secondary cell walls of individual cotton fibers remain unclear. A better understanding of these mechanisms would facilitate the development of cotton cultivars with higher quality fibers.

The genetic and molecular basis of the traits of high quality fibers can be effectively investigated with genome-wide transcriptome profiles of relevant genes and pathways (Tuttle *et al.* 2015; Fang *et al.* 2014a,b); transcriptome analyses have provided insight into the dynamic regulation of fiber development (Tu *et al.* 2007; Islam *et al.* 2016b; Ma *et al.* 2016; Hu *et al.* 2013; Liang *et al.* 2015). Indeed, recent comparative transcriptomes analyses have suggested several genes as candidates for molecular breeding, genetic manipulation, and the regulation of cell-wall assembly and strength (Islam *et al.* 2016b; Wu *et al.* 2016). However, few studies have focused on introgression lines carrying *G. barbadense* fragments in a *G. hirsutum* background (Fang *et al.* 2014a,b).

To investigate the molecular mechanisms underlying cotton fiber strength regulation and the production of superior quality fibers, with a particular focus on introgression lines, we first aimed to create a population of CSSLs. We then aimed to identify CSSLs with higher and lower fiber strength than the recurrent parent, and sequence their transcriptomes. Finally, we aimed to compare, in developing fibers, the genes expressed differently in the higher fiber strength CSSLs as compared to the lower fiber strength CSSLs. We hypothesized that genes associated with different metabolic pathways would affect fiber strength and, in different introgression lines, gene expression in the same pathway would change over time.

## Materials and Methods

### Genotyping and fiber strength testing

At the Institute of Cotton Research of the Chinese Academy of Agricultural Sciences (CRICAAS; Anyang, Henan province, China), we derived a population of 332 CSSLs by backcrossing the donor parent Hai1 with the recurrent parent CCRI45 for four generations, followed by three consecutive generations of selfing. Hai1 is a conventional cultivar of *G. barbadense* with a dominant glandless gene, high resistance to *Verticillium* wilt, and excellent fiber quality (Shi *et al.* 2015). CCRI45 is a widely grown upland cotton cultivar with high yield developed by CRICAAS (Shi *et al.* 2015). The 332 CSSLs and the two parent cultivars were genotyped. We then tested fiber strength across three environments: Anyang, China (2010, 2011), and Aksu, Xinjiang Uyghur Autonomous Region (2011; Supplemental Material, Figure S1; (Shi *et al.* 2015; Ma 2013; B. Li *et al.* 2016). Based on these results, we selected two CSSLs with significantly higher fiber strength than the recurrent parent CCRI45, and one with lower fiber strength. Using the previously constructed genetic linkage map (Shi *et al.* 2015), we selected 526 pairs of simple sequence repeats (SSRs) at intervals of 10–20 cM to screen for introgressed *G. barbadense* chromosomal segments and polymorphisms between CCRI45 and each of the three selected CCSLs. 

### Planting and harvesting

The selected CSSLs as well as the recurrent parent CCRI45 were planted at the Experimental Farm at CRICAAS in 2014. We tagged all flowers on the day of anthesis, and harvested 3–10 bolls from the tagged flowers by 10:00 am at 15, 20, 25, and 28 DPA. Harvested bolls were immediately immersed in ice. To minimize experimental error, all samples from the same developmental stage were tagged and collected on the same day. Fiber samples from the ovules of 15–28 developing bolls were dissected, frozen into liquid nitrogen, and stored at −70°. We sampled each developmental stage of each genotype three times.

### RNA isolation and calibration

We isolated total RNA from each fiber sample replicate heavier than 1 g using a modified CTAB extraction protocol (Jiang and Zhang 2003): a total of 45 fiber samples. After RNA extraction, RNA degradation and contamination were monitored on 1% agarose gels. We measured RNA purity with a NanoPhotometer spectrophotometer (IMPLEN, CA); RNA concentration with a Qubit RNA Assay Kit (Life Technologies, CA) using a Qubit 2.0 Fluorometer (Life Technologies); and RNA integrity with a RNA Nano 6000 Assay Kit (Agilent Technologies, CA) using a Bioanalyzer 2100 (Agilent Technologies). Equal aliquots of the RNA isolated from the each of the three replicates were mixed to yield one total RNA sample from each time point.

### Transcriptome sequencing

To systematically analyze transcriptome profiles relating to cotton fiber development, we first constructed sequence libraries with a NEBNext Ultra RNA Library Prep Kit for Illumina (NEB) following the manufacturer’s instructions; index codes were added to attribute sequences to each sample. Briefly, 150 ng of each total RNA sample was sheared, converted to cDNA, and ligated to sequencing adaptors following the manufacturer’s protocol. The library fragments were purified using AMPure XP (Beckman Coulter, Beverly). We preferentially selected 150–200 bp fragments. We constructed a total of 15 cDNA libraries from the total RNA samples: four collected at 15 DPA, four collected at 20 DPA, four collected at 25 DPA, and three collected at 28 DPA. Transcriptomes were sequenced on an Illumina Hiseq2000 V4 by Berry Genomics Co., Ltd. (Beijing, China), and 125 bp paired-end reads were generated.

### Data processing and statistical evaluation

Sequences were filtered and cleaned with the NGS QC Toolkit (http://www.nipgr.res.in/ngsqctoolkit.html; (Patel and Jain 2012). Cleaned sequences were mapped to the reference genome of *G. hirsutum* (T. Zhang *et al.* 2015) with TopHat v2 (http://ccb.jhu.edu/software/tophat/index.shtml; (Kim *et al.* 2013), allowing single mismatches but discarding multiple mismatches. Gene expression levels are subject to transcript abundance, but this can be controlled by the calculation of reads mapped to reference genes or exons (Anders and Huber 2010). Thus, expression levels are expressed as reads per kilobase per million mapped reads (RPKM).

### Cluster and functional analyses

We calculated the Pearson correlation coefficient (PCC) for all genes. We used the “Self-Organizing Tree Algorithm” (SOTA; MeV 4.9.0; (Wang *et al.* 1998) to cluster the log2-transformed RPKM values of all genes at 15, 20, 25, and 28 DPA. We clustered CSSL genes in CSSLs at 15, 20, 25, and 28 DPA with Cluster3.0 (http://bonsai.hgc.jp/∼mdehoon/software/cluster/software.htm; (de Hoon *et al.* 2004). Analysis of gene ontology (GO) was performed using BLAST2GO (http://www.blast2go.com/b2ghome; (Conesa *et al.* 2005) with *E*-value set to 1E-5 and max_target_seqs set to 20. We used Fisher’s exact test to test for statistically significant differences, and the Benjamini and Hochberg false discovery rate (FDR) procedure to adjust for the effects of multiple tests (Benjamini *et al.* 2001). GO terms were considered significant when FDR < 0.01.

### Selection of DEGs

To identify the DEGs in the secondary cell wall formation stage of fiber development, gene expression levels of all three CSSLs were compared with those of the recurrent parent CCRI45 at 15, 20, 25, and 28 DPA. DEGs were identified using the generalized fold change algorithm (GFOLD, http://www.tongji.edu.cn/∼zhanglab/GFOLD/index.html; Feng *et al.* 2012). Genes with a GFOLD value ≥0.5 were considered significantly upregulated DEGs, and genes with GFOLD value ≤−0.5 were considered significantly downregulated DEGs. DEGs found in both high quality lines (MBI7747 and MBI7561) but not in the low-quality line (MBI7285) were defined as common DEGs. We considered DEGs where |Gfold value|≥ 1 “significant DEGs.” To better understand the different gene expression patterns in the three introgression lines as compared with the recurrent parent CCRI45, we identified the significant DEGs (|Gfold value|≥ 1) found in each CSSL from the 2200 common DEGs, and compared the number of significant DEGs found in each CSSL to the number found in the recurrent parent CCRI45. We next focused on the functions of the genes differently expressed by MBI17561, clustering the DEGs following the procedure outlined above. In addition, we performed a Venn analysis with the SAB lab web tool (http://sablab.net/venn.php; Yang *et al.* 2010).

### DEGs associated with fiber strength

Phytohormones play an important regulatory role in various plant growth and developmental processes, factors directly related to fiber strength (Sun *et al.* 2015). Transcription factors also regulate fiber strength (Salih *et al.* 2016). We identified common DEGs associated with these processes (*e.g.*, hormone signal transduction and biosynthesis of auxin, brassinosteroid, and ethylene) and integrated the expression profiles. We then organized these DEGs by biological process. Next, to study changes in the biological processes identified in MBI7561 but absent in CCRI45, we identified DEGs involved in the same biological processes across at least two time points. We used BLASTP to search for proteins encoded by these genes.

### Colocalization of common DEGs

Based on the genomic position of the introgressed *G. barbadense* chromosome segments identified above, we identified the common DEGs in those introgressed regions from the transcriptome data using the gene name. We annotated the colocalized common DEGs with GO as detailed above.

### Quantitative real-time PCR (qRT-PCR)

To test the reliability of transcriptome data and to validate the gene expression data, we performed qRT-PCR analyses on three DEGs that were not only expressed in MBI7561 across all four time points, but were also differentially expressed between at least two of those time points. In addition, we used seven genes selected randomly. We performed qRT-PCR on all DEGs with a 7500 Fast Real-Time PCR System (Applied Biosystems, San Francisco, CA). We designed gene-specific primers with Primer Premier 5.0 (Premier BioSoft International, Palo Alto, CA) and checked their specificity with BLAST (https://blast.ncbi.nlm.nih.gov/Blast.cgi; Altschul *et al.* 1990). Our novel primers (Table S1) were synthesized by Sangon Biotech Co., Ltd. (Shanghai, China). All DEGs were analyzed three times. We used the histone3 gene of *G. hirsutum* (forward primer: 5′-GGTGGTGTGAAGAAGCCTCAT-3′; reverse primer: 5′-AATTTCACGAACAAGCCTCTGGAA-3′) as the internal reference. Relative gene expression levels were calculated as 2^−ΔCT^.

### Data availability

The data sets supporting the results of this article are included within the article and its Supplemental Materials. Figure S1, development of the CSSLs. Figure S2, graphical representation of CSSL genotypes. Figure S3, GO of the common DEGs anchored to quantitative trait loci. Table S1, the specific primer sequences. Table S2, annotation of genes differentially expressed. Table S3, the common DEGs located in common introgressed *G. barbadense* chromosome segments.

## Results

### CSSLs genotypes and fiber strength

The mean fiber strengths of two of the selected CSSLs, MBI7561 (34.30 cN/tex) and MBI7747 (36.50 cN/tex), were significantly higher than that of the recurrent parent CCRI45 (27.97 cN/tex), while the mean strength of the third selected CSSL, MBI7285 (25.90 cN/tex), was lower (Table 1). Our CSSLs contained some chromosomal segments of *G. barbadense* in a *G. hirsutum* background. The introgressed *G. barbadense* chromosomal segments were different in each of the three CSSLs. Among the 526 SSRs screened, we found 27 polymorphisms in MBI7561, 11 in MBI7747, and 39 in MBI7285 as compared to CCRI45 (Figure S2 and Table 1): <7.41% of the total chromosome.

**Table 1:** Polymorphism and fiber strength of the three CSSLs and the recurrent parent CCRI45

Cotton	Number of Differential Makers	Proportion of Polymorphic Markers*^a^*	Upper Half Mean Length (mm)	Mironaire	Fiber Strength (cN/tex)
MBI7561	27	5.13%	32.10 ± 1.65*^b^*	5.10 ± 045	34.30 ± 1.89*^b^*
MBI7747	11	2.10%	33.80 ± 1.64*^b^*	4.30 ± 0.41	36.50 ± 1.83*^b^*
MBI7285	39	7.41%	27.10 ± 1.34	4.40 ± 0.45	25.90 ± 1.76
CCRI45	0	0	28.13 ± 0.71	5.03 ± 0.49	27.97 ± 0.93

a 526 SSR markers used for genetic testing.

b CSSLs significantly different from CCRI45 (*P* < 0.01).

### Functional clusters

**Figure 1:**
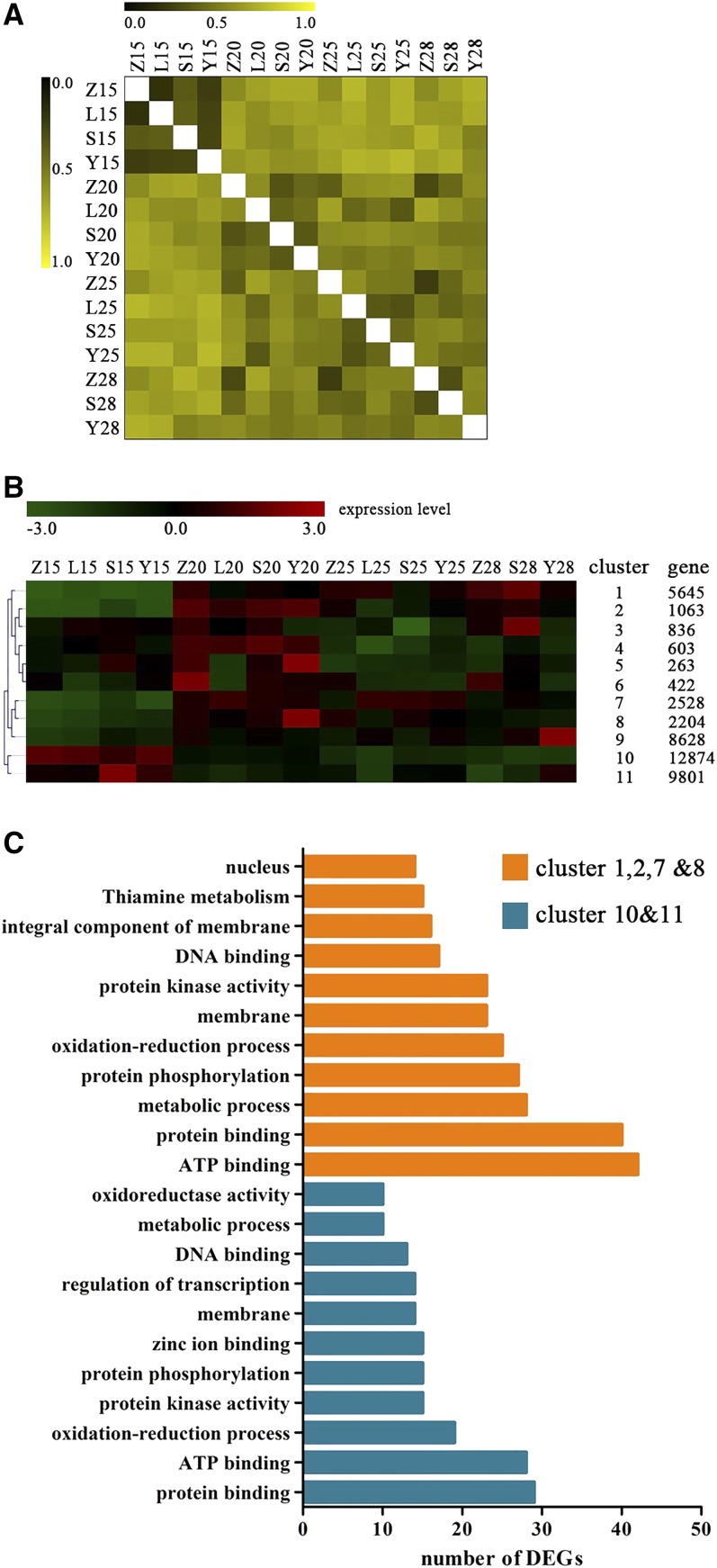
Statistical analysis of transcript profiling data. Z, S, L, and Y indicate CCRI45, MBI7561, MBI7747, and MBI7285, respectively. 15, 15 DPA; 20, 20 DPA; 25, 25 DPA; and 28, 28 DPA. (A) Pearson correlation coefficients of all samples. (B) Self-Organizing Tree Algorithm clustering of all genes across all samples. (C) Functional annotations of genes in more highly expressed at 15 DPA than later (orange bars) and those more highly expressed at 28 DPA than earlier (blue bars). DEG, differentially-expressed gene; DPA, days postanthesis.

All the raw data we sequenced was deposited in the NCBI short read archives (SRA; accession number SRP084203). We identified 44,687 genes across all cDNA libraries. The PCC was low for CCRI45 at all DPAs tested, and was also low for all three CSSLs at 15 DPA. However, higher PCCs were observed at 20 DPA, 25 DPA, and 28 DPA (Figure 1A). The 44,678 identified genes were grouped into 11 clusters based on expression patterns (Figure 1B). Genes in clusters 10 and 11 were more highly expressed at 15 DPA than at later stages, while genes in clusters 1, 2, 7, and 8 had the opposite pattern (Figure 1B). GO analysis suggested that genes in all six of these clusters shared some similar functions, such as the regulation of protein and ATP binding, but differed substantially as well: genes in clusters 10 and 11 were integral components of membrane and thiamine metabolisms, while genes in clusters 1, 2, 7, and 8 were mainly related to the regulation of transcription (*e.g.*, zinc ion binding; Figure 1C).

### Analysis of common DEGs

**Figure 2:**
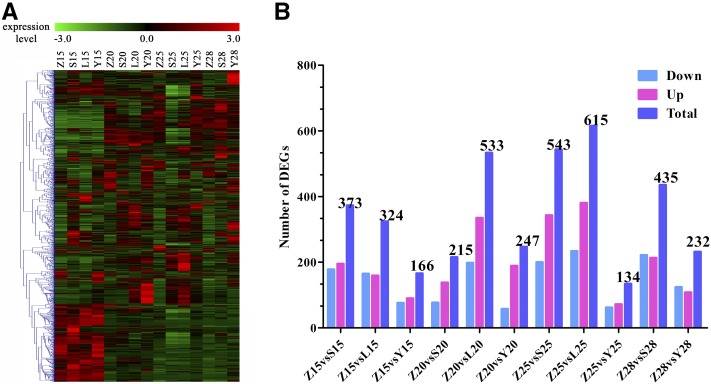
Overview of DEGs. Z, S, L, and Y indicate CCRI45, MBI7561, MBI7747, and MBI7285, respectively. 15, 15 DPA; 20, 20 DPA; 25, 25 DPA; and 28, 28 DPA. (A) Heatmap of all common DEGs at 15, 20, 25, and 28 DPA. (B) Expression pattern of common DEGs over the four time points as compared to the recurrent parent CCRI45. DEG, differentially-expressed gene; DPA, days postanthesis.

**Figure 3:**
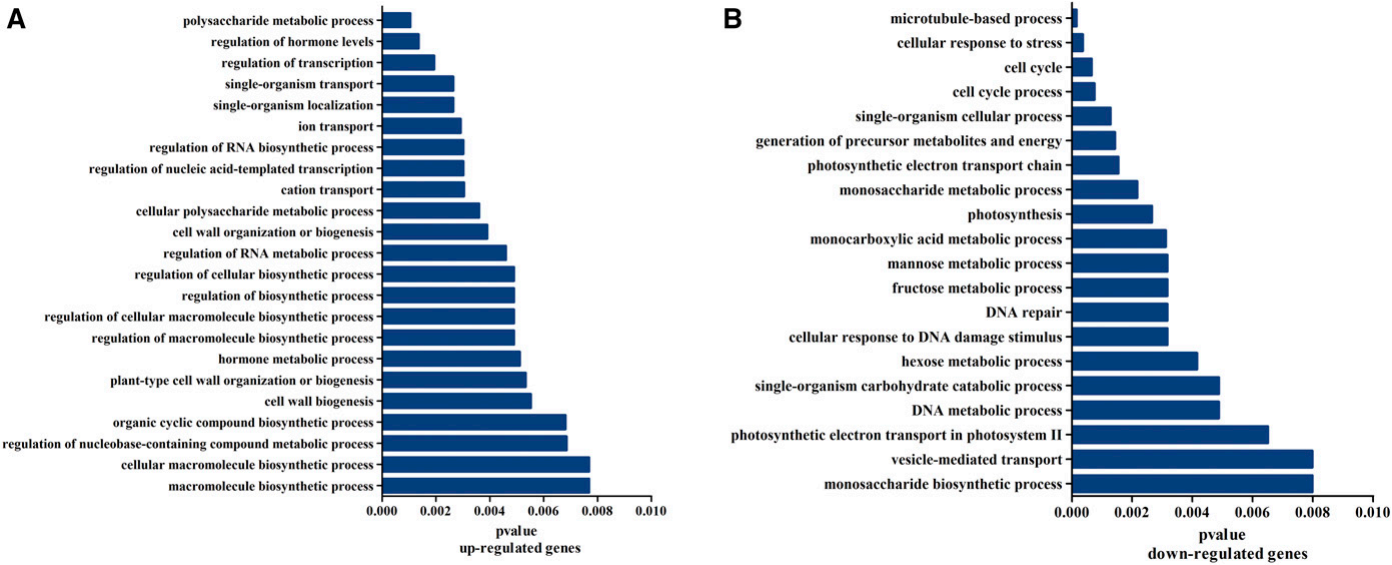
Functional associations of common differentially-expressed genes (DEGs). (A) Biological processes associated with upregulated common DEGs. (B) Biological processes associated with downregulated common DEGs.

In total, we obtained 2200 common DEGs. Most of these were expressed at low levels (Figure 2A). More significant DEGs were identified in MBI7747 at 15, 20, and 25 DPA than in CCRI45 (Table 2). When compared to CCI45 at 25 DPA, 615 significant DEGs were identified in MBI7747 and 543 were identified in MBI7561, many more than were identified in MBI7285 (Figure 2B). The common DEGs that were significantly upregulated (GFOLD > 0.5) were associated with polysaccharide metabolic processes, regulation of hormone levels, and regulation of transcription (Figure 3A). The common DEGs that were significantly downregulated (GFOLD < 0.5) were associated with microtubule-based processes, cellular response to stress, and the cell cycle (Figure 3B).

**Table 2:** All significant DEGs in diverse comparisons

Comparison	Down	Up	Total
CCRI45-15DPA *vs.* MBI7561-15DPA	178	195	373
CCRI45-15DPA *vs.* MBI7747-15DPA	165	159	324
CCRI45-15DPA *vs.* MBI7285-15DPA	76	90	166
CCRI45-20DPA *vs.* MBI7561-20DPA	77	138	215
CCRI45-20DPA *vs.* MBI7747-20DPA	198	335	533
CCRI45-20DPA *vs.* MBI7285-20DPA	58	189	247
CCRI45-25DPA *vs.* MBI7561-25DPA	200	343	543
CCRI45-25DPA *vs.* MBI7747-25DPA	234	381	615
CCRI45-25DPA *vs.* MBI7285-25DPA	62	72	134
CCRI45-28DPA *vs.* MBI7561-28DPA	222	213	435
CCRI45-28DPA *vs.* MBI7285-28DPA	124	108	232

DPA, days postanthesis.

### Cluster analysis of significant DEGs in high fiber strength CSSLs

**Figure 4:**
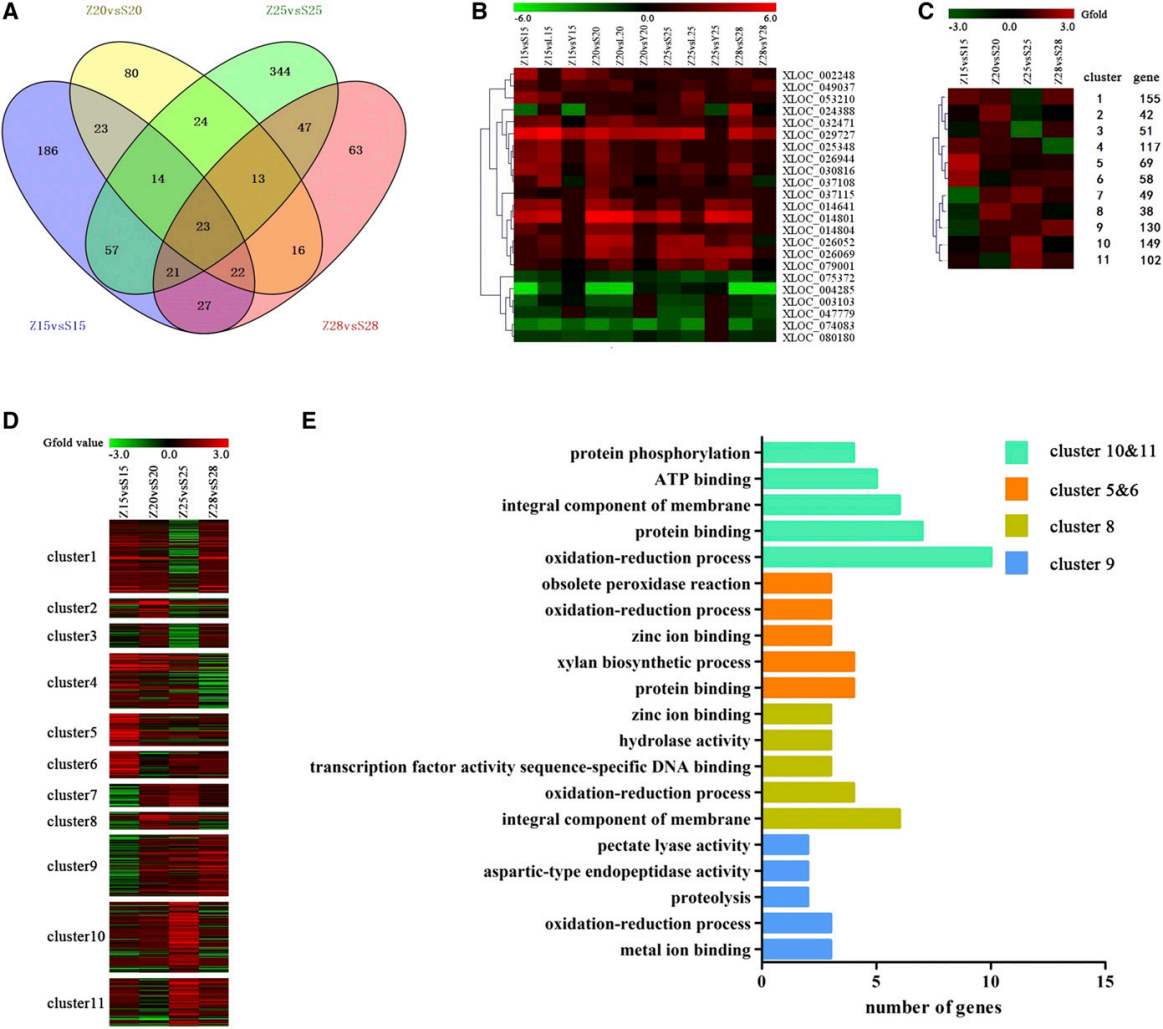
Cluster analysis of the common DEGs. Z, S, L, and Y indicate CCRI45, MBI7561, MBI7747, and MBI7285, respectively. 15, 15 DPA; 20, 20 DPA; 25, 25 DPA; and 28, 28 DPA. (A) Venn diagram of significant DEGs found in MBI7561 and CCRI45 across various time points. (B) Heatmap of common DEGs. (C) Self-Organizing Tree Algorithm clustering of all significant DEGs between CCRI45 and MBI7561 at all four time points. (D) Heatmap analysis of the expression of all significant DEGs between CCRI45 and MBI7561 at all four time points. (E) Functional annotations of significant DEGs: green bars indicate genes highly expressed at 15 DPA; orange bars indicate genes highly expressed at 20 DPA; gold bars indicate genes highly expressed at 25 DPA; and blue bars indicate genes highly expressed at 28 DPA. ATP, adenosine triphosphate; DEG, differentially-expressed gene; DPA, days postanthesis.

Our Venn analysis indicated 23 common DEGs to be significantly up- or downregulated in MBI7561 but not CCRI45 at 15, 20, 25, and 28 DPA (Figure 4A). These same 23 common DEGs were also significantly up- or downregulated in MBI7747 but not CCRI45 at 15, 20, and 25 DPA (28 DPA not available; Figure 4B). Over all four time points, we found 960 genes that were significantly up- or downregulated in MBI7561 as compared to CCRI45. These 960 significant DEGs were sorted into 11 clusters by expression pattern (Figure 4C). Genes in clusters 5 and 6 (regulation of protein binding and xylan biosynthesis) were highly expressed at 15 DPA; genes in cluster 8 (regulation of integral components of membranes and oxidation–reduction processes) were highly expressed at 20 DPA; genes in clusters 10 and 11 (regulation of oxidation–reduction processes and protein binding) were highly expressed at 25 DPA; and genes in cluster 9 (regulation of metal ion binding and oxidation–reduction processes) were highly expressed at 28 DPA (Figure 4, D and E).

### Common DEGs related to fiber strength

**Figure 5:**
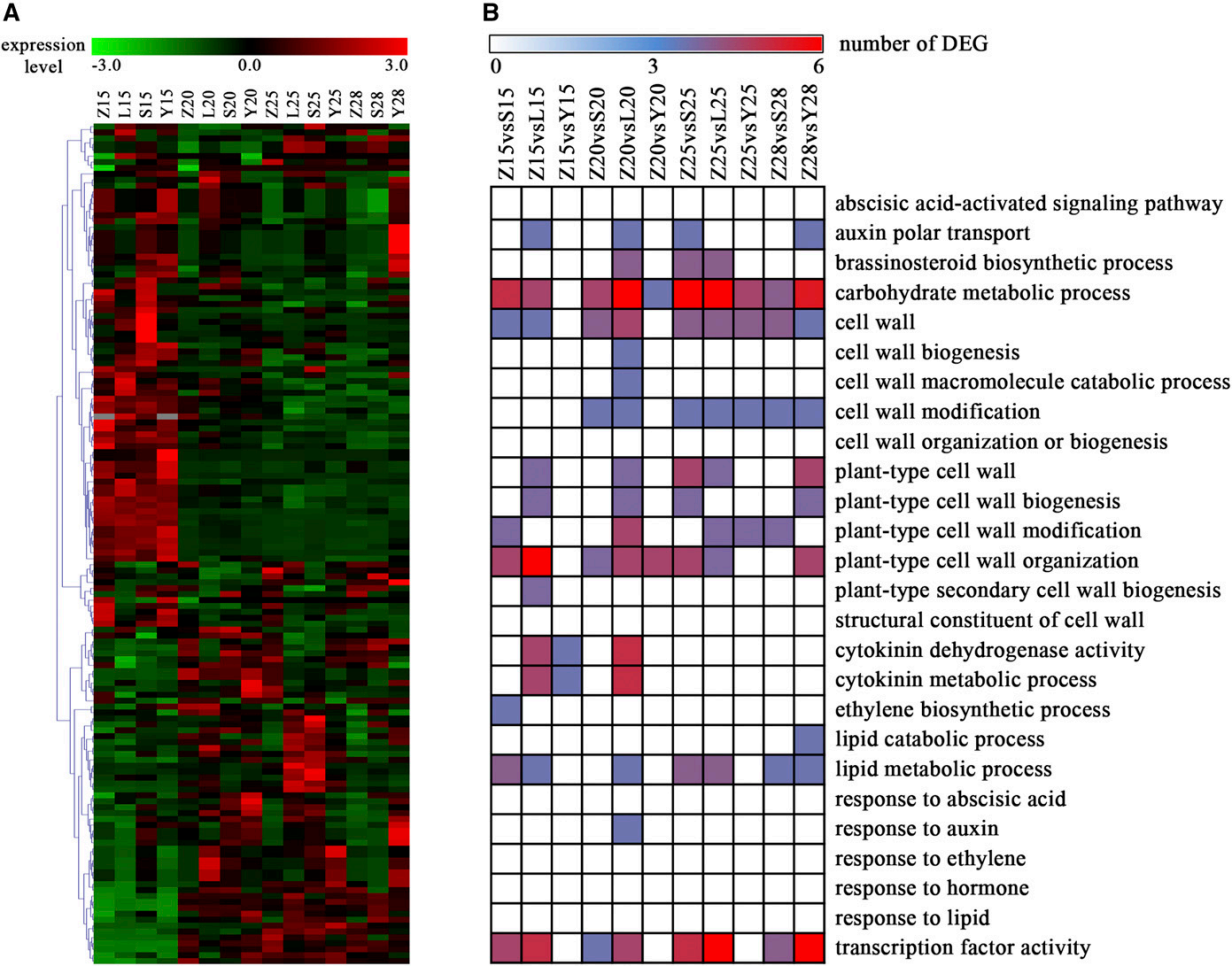
Common DEGs related to fiber strength. Z, S, L, and Y indicate CCRI45, MBI7561, MBI7747, and MBI7285, respectively. 15, 15 DPA; 20, 20 DPA; 25, 25 DPA; and 28, 28 DPA. (A) Heatmap of common DEGs related to fiber strength. (B) Numbers of common DEGs involved in processes related to fiber strength. DEG, differentially-expressed gene; DPA, days postanthesis.

We found 31 common DEGs associated with phytohormones: 18 in hormone signal transduction and 13 in the biosynthesis of auxin, brassinosteroid, and ethylene. In addition, 75 common DEGs involved in cell wall and fatty acid biosynthesis were observed. Finally, 59 DEGs were associated with carbohydrate metabolism. Our results indicated that most of the genes associated with these biological processes were minimally expressed (Figure 5A). After sorting by biological process, it was clear that most DEGs regulated the cell wall, carbohydrate metabolism, and transcription factor activation (Figure 5B).

### Colocalization of common DEGs

We colocalized 16 common DEGs in two *G. barbadense* chromosome segments, present in both MBI7561 and MBI7747. Among these 16 DEGs, 10 were downregulated and 6 were upregulated; four were associated with cellular processes, five were associated with single-organism processes, four were associated with catalytic activity, and four were associated with the membrane and the membrane-enclosed lumen (Figure S3).

### Validation of DEGs with qRT-PCR

**Figure 6:**
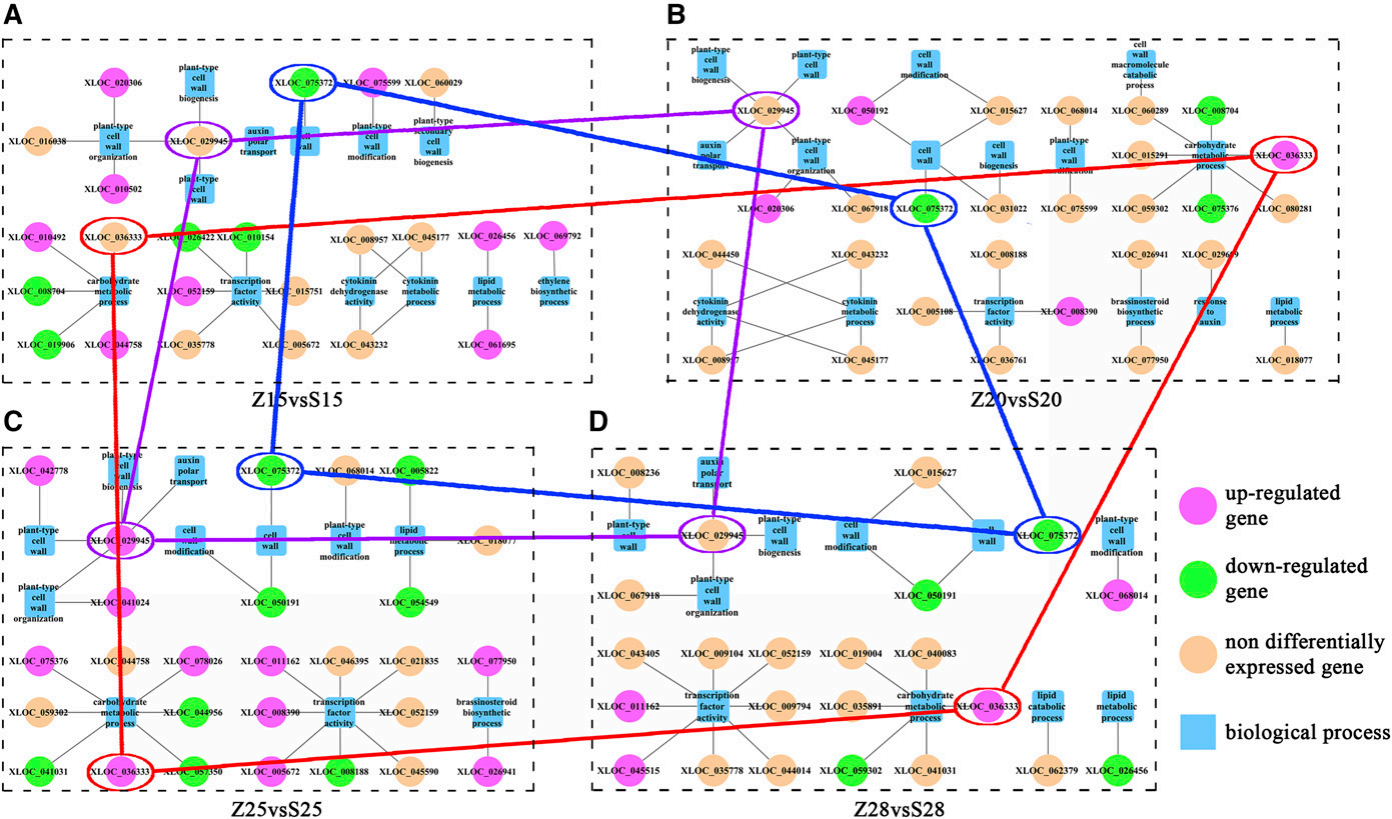
Biological functions associated with selected DEGs in MBI7561. Z, S, L, and Y indicate CCRI45, MBI7561, MBI7747, and MBI7285, respectively. 15, 15 DPA; 20, 20 DPA; 25, 25 DPA; and 28, 28 DPA. (A) Biological processes of genes at 15 DPA. (B) Biological processes of genes at 20 DPA. (C) Biological processes of genes at 25 DPA. (D) Biological processes of genes at 28 DPA. DEG, differentially-expressed gene; DPA, days postanthesis.

We selected three DEGs expressed in MBI7561 but absent in MBI7285 at all four time points, with differing expression for at least two time points: XLOC_036333, XLOC_029945, and XLOC_075372 (Figure 6). XLOC_036333 [encodes *MNS1*] was upregulated at 20, 25, and 28 DPA, while XLOC_029945 (encodes fasciclin-like arabinogalactan protein 8, FLA8) was upregulated only at 25 DPA. XLOC_075372 (encodes *snakin-1*) was downregulated at all time points.

**Figure 7:**
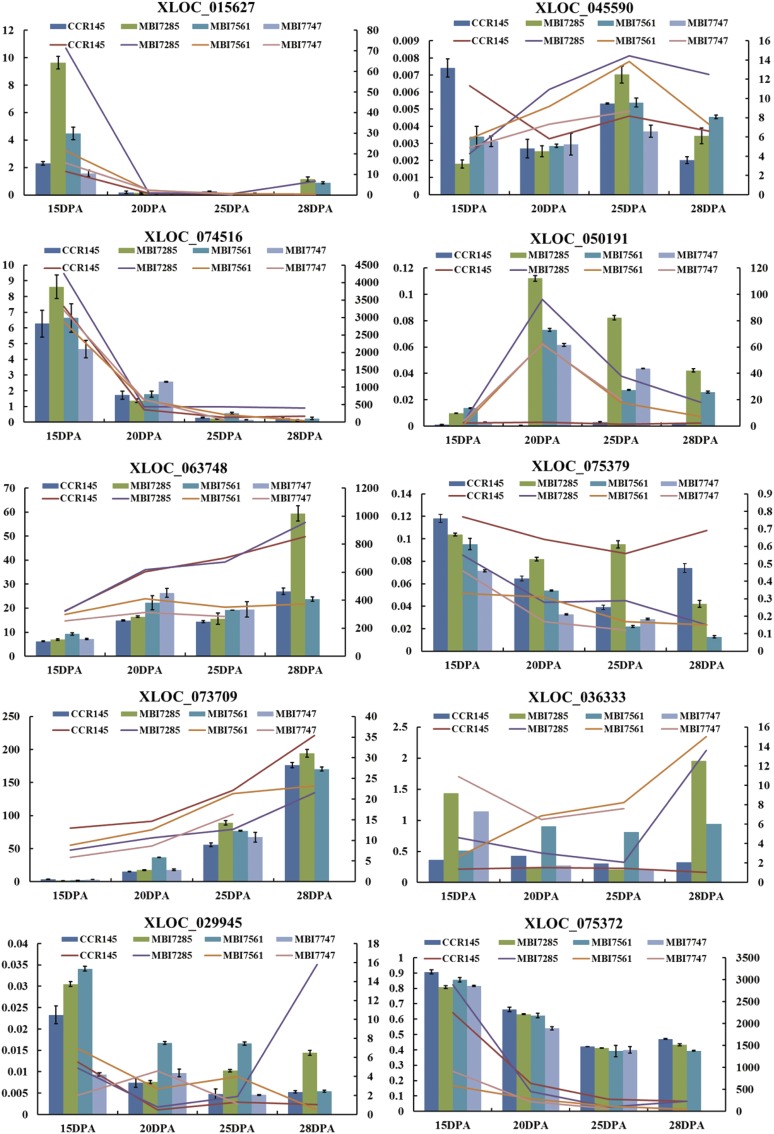
Quantitative real-time polymerase chain reaction validation of 10 DEGs. DPA, days postanthesis.

The DEG expression patterns recovered by our qRT-PCR analysis of the three DEGs identified above (XLOC_036333, XLOC_029945, and XLOC_075372) and the seven other randomly selected genes were consistent with the results of transcriptome data (Figure 7).

## Discussion

Cotton fiber strength, primarily determined during the secondary cell wall formation stage, is a critical component of fiber quality (Lee *et al.* 2007). Genetic screening has identified a number of genes involved in fiber development (Z. Zhang *et al.* 2015), but the mechanisms by which these genes affect fiber strength and development remain poorly understood. Recently, several large-scale genomic studies have identified genes that are associated with cotton fiber development and involved in related signaling pathways (Yang *et al.* 1982; Liang *et al.* 2015; Naoumkina *et al.* 2015; Fang *et al.* 2014a,b). Transcriptome analyses suggested that gene expression patterns and functional distributions vary significantly across different stages of fiber development due to the complexities of the mechanisms involved and the regulatory components in the process (Fang *et al.* 2014a,b; Islam *et al.* 2016b; Tuttle *et al.* 2015).

We found 2200 common DEGs in the two high fiber strength CSSLs, as compared to the recurrent parent CCRI45 (Figure 2B), indicating differences in gene expression patterns between the CSSLs and the recurrent parent. Additionally, these common DEGs clustered into functional groups that were modulated differently over time (Figure 1A), suggesting that gene expression profiles in the same pathway, as well as the genes involved in that pathway, may be altered differently at various stages of fiber development. We identified several functional candidate genes involved in secondary cell wall formation, mainly related to biological processes such as polysaccharide metabolism, regulation of hormone levels, regulation of transcription, and cell wall biogenesis (Figure 1C). Thus, consistent with our hypothesis, our results indicated that genes associated with different metabolic pathways affect fiber strength.

Most of the 2200 common DEGs identified in this study are involved in cell wall biogenesis. We propose the DEGs that we identified as being implicated in secondary wall synthesis and metabolism as candidates for the further development of cotton cultivars with superior fiber quality. However, genes involved in the regulation of polysaccharide metabolic processes were also upregulated in our high fiber strength CSSLs. It has been shown that polysaccharide components such as pectin also play an important role in secondary cell wall synthesis (Gou *et al.* 2007), and that an increase in noncellulosic polysaccharides may trigger a delay in the transition to secondary wall synthesis, resulting in improved fiber quality (Y. Li *et al.* 2016).

We also recovered several common DEGs involved in hormone signal transduction and the biosynthesis of auxin, brassinosteroid, and ethylene. Previous studies have suggested that ethylene might promote cell elongation by increasing the expression of sucrose synthase, tubulin, and expansin genes (Shi *et al.* 2006); activation of ethylene biosynthesis by saturated very-long-chain fatty acids significantly promoted fiber cell elongation in *G. hirsutum* (Qin *et al.* 2007).

Our colocalization indicated that 16 of the common DEGs were located segments known to harbor quantitative traits for fiber strength (Ma 2013), further suggesting a relationship between these common DEGs and fiber strength.

Based on these analyses, we selected three genes putatively associated with fiber strength, expressed in MBI7561 at all four time points. *FLA8* (XLOC_029945) was upregulated at 25 DPA in the high fiber strength CSSLs, suggesting that *FLA8* may have a tissue-specific expression preference for the secondary cell wall developmental stage, and may contribute to fiber strength by affecting cellulose synthesis and the orientation of microfibril deposition. *FLA8* is involved in moderating and organizing the cell wall, anchoring components of the plasma membrane, regulation of cell size, and auxin polar transport (Lee *et al.* 2005). *FLA8* has been previously described as a cell wall-associated protein regulating plant growth and development, including cell wall pectin plasticization, cell expansion, and cell wall architecture (Lee *et al.* 2005); *FLA8* might play a critical role in the regulation of fiber strength (Liu *et al.* 2013). Genes encoding *FLA8* were differentially expressed across various plant tissues, in developing fibers, and between *G. hirsutum* and *G. barbadense* (Pan *et al.* 2007; Liu *et al.* 2008, 2013).

The gene *snakin-1* (XLOC_075372), which was downregulated at all time points, regulates the extracellular region, cell wall, and defense response (Nahirnak *et al.* 2012). The silencing of *snakin-1* has been reported to affect cell division, primary leaf metabolism, and cell wall composition in potato plants (Nahirnak *et al.* 2012). Therefore, the downregulation of *snakin-1* we observed indicates that it may play an important role in fiber strength by affecting the composition and structure of the cell wall.

The gene *MNS1* (XLOC_036333) was upregulated at 20 and 28 DPA. This gene is likely to be related to fiber strength regulation as it encodes an enzyme belonging to the glycosyl hydrolase family, involved in the synthesis of glycoproteins (Zhou *et al.* 2012). It also regulates mannosyl-oligosaccharide 1,2-α-mannosidase activity, membrane and calcium ion binding, and carbohydrate metabolism (Hosomi *et al.* 2010). Therefore, these three genes might regulate important biological processes involved in cell wall biogenesis and might mediate secondary cell wall deposition, contributing to the development of cotton fiber strength. As such, these three genes may be particularly good candidates for future investigation of the molecular mechanisms of fiber strength formation, and for the improvement of cotton fiber quality through molecular breeding.

## 

## Supplementary Material

Supplemental material is available online at www.g3journal.org/lookup/suppl/doi:10.1534/g3.117.300108/-/DC1.
