# Supplementary figures and images for: Transcriptome Analysis Suggests That Chromosome Introgression Fragments from Sea Island Cotton (Gossypium barbadense) Increase Fiber Strength in Upland Cotton (Gossypium hirsutum)
Source: G3 (Bethesda). 2017 Sep 5;7(10):3469–79. doi: 10.1534/g3.117.300108 (PMC5633395; doi:10.1534/g3.117.300108)

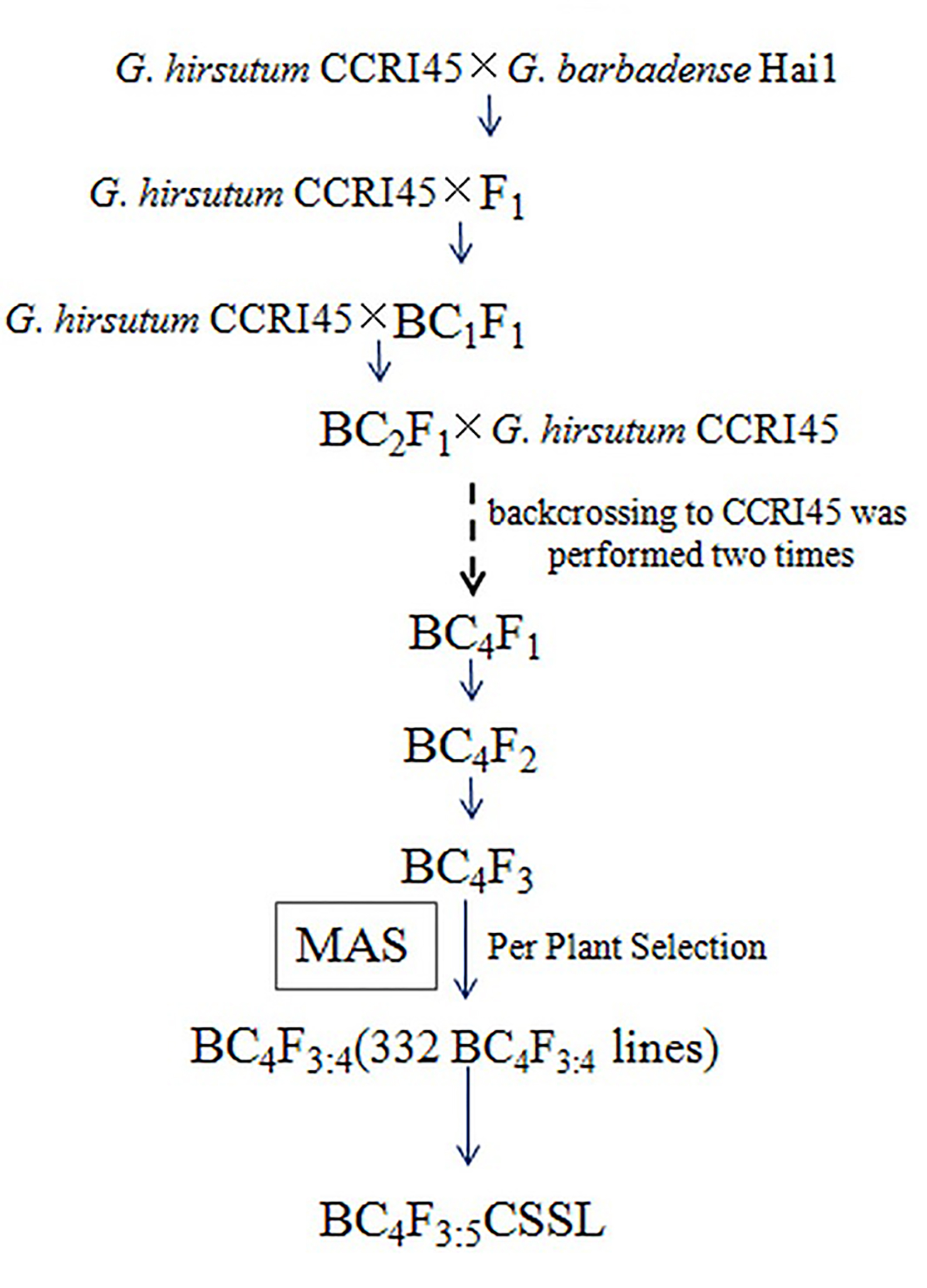

Supplement: Supplementary file 1 [file 3469FigureS1.jpg]

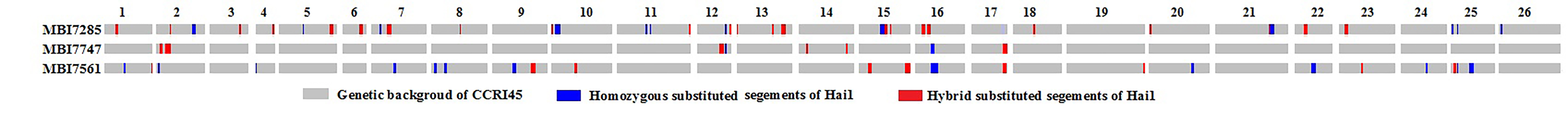

Supplement: Supplementary file 2 [file 3469FigureS2.jpg]
