# Supplementary material for: Transcriptome Analysis Suggests That Chromosome Introgression Fragments from Sea Island Cotton (Gossypium barbadense) Increase Fiber Strength in Upland Cotton (Gossypium hirsutum)
Source: G3 (Bethesda). 2017 Sep 5;7(10):3469–79. doi: 10.1534/g3.117.300108 (PMC5633395; doi:10.1534/g3.117.300108)
Supplement: Supplementary file 7 [file 3469FileS1.docx]

Figure S1. Development of the chromosome segment substitution lines (CSSLs).

Figure S2. Graphical representation of chromosome segment substitution line (CSSL) genotypes.

Figure S3. Gene ontology of the common differentially expressed genes anchored to quantitative trait loci.

Table S1. The specific primer sequences.

Table S2. Annotation of genes differentially expressed.

Table S3. The common DEGs located in common introgressed *G. barbadense* chromosome segments.
